# Comparative proteomic analysis reveals that the Heterosis of two maize hybrids is related to enhancement of stress response and photosynthesis respectively

**DOI:** 10.1186/s12870-020-02806-5

**Published:** 2021-01-09

**Authors:** Daoping Wang, Yongying Mu, Xiaojiao Hu, Bo Ma, Zhibo Wang, Li Zhu, Jiang Xu, Changling Huang, Yinghong Pan

**Affiliations:** 1 Institute of Crop Sciences, Chinese Academy of Agricultural Sciences, Beijing, 100081 People’s Republic of China; 2 National Engineering Laboratory for Crop Molecular Breeding, Beijing, 100081 People’s Republic of China; 3 Xiyuan Hospital, China Academy of Chinese Medical Sciences, Beijing, 100091 People’s Republic of China; 4 Biotechnology Research Institute, Chinese Academy of Agricultural Sciences, Beijing, 100081 People’s Republic of China; 5 National Key Facility for Crop Gene Resources and Genetic Improvement, Beijing, 100081 People’s Republic of China

**Keywords:** Heterosis, Non-additive protein, Proteomic analysis, Maize

## Abstract

**Background:**

Heterosis refers to superior traits exhibiting in a hybrid when compared with both parents. Generally, the hybridization between parents can change the expression pattern of some proteins such as non-additive proteins (NAPs) which might lead to heterosis. ‘Zhongdan808’ (ZD808) and ‘Zhongdan909’ (ZD909) are excellent maize hybrids in China, however, the heterosis mechanism of them are not clear. Proteomics has been wildly used in many filed, and comparative proteomic analysis of hybrid and its parents is helpful for understanding the mechanism of heterosis in the two maize hybrids.

**Results:**

Over 2000 protein groups were quantitatively identified from second seedling leaves of two hybrids and their parents by label-free quantification. Statistical analysis of total identified proteins, differentially accumulated proteins (DAPs) and NAPs of the two hybrids revealed that both of them were more similar to their female parents. In addition, most of DAPs were up-regulated and most of NAPs were high parent abundance or above-high parent abundance in ZD808, while in ZD909, most of DAPs were down-regulated and most of NAPs were low parent abundance or below-low parent abundance. Pathway enrichment analysis showed that more of stress response-related NAPs in ZD808 were high parent abundance or above-high parent abundance, and most of PS related NAPs in ZD909 were high parent abundance or above-high parent abundance. Finally, four stress response-related proteins and eight proteins related to PS were verified by PRM, ten of them had significant differences between hybrid and midparent value.

**Conclusions:**

Even though every one of the two hybrids were more similar to its female parent at proteome level, the biological basis of heterosis is different in the two maize hybrids. In comparison with their parents, the excellent agronomic traits of hybrid ZD808 is mainly correlated with the high expression levels of some proteins related to stress responses and metabolic functions, while traits of ZD909 is mainly correlated with high expressed proteins related to photosynthesis. Our proteomics results support previous physiological and morphological research and have provided useful information in understanding the reason of valuable agronomic traits.

**Supplementary Information:**

The online version contains supplementary material available at 10.1186/s12870-020-02806-5.

## Highlights

- Nearly 200 NAPs were identified in maize hybrids ZD808 and ZD909 respectively.
- Most heterosis related proteins and pathways were different between two hybrids.
- Pathways related to stress response were significantly enhanced in hybrid ZD808.
- Pathways related to photosynthesis were significantly enhanced in hybrid ZD909.

## Background

Heterosis refers to the phenomenon that of superior traits that exhibiting in hybrid progeny of species or varieties compared with both parents. This can include important agronomic traits, such as biomass, developmental speed, fertility, yield, and abiotic/biotic stress tolerance. Thus, F1 hybrids displaying heterosis are widely used in the production of many crops and vegetables [1, 2]. However, to obtain a hybrid with heterosis, a large number of field tests are needed, which are labor intensive, expensive, time consuming and long-term processes, and the results are unpredictable. To improve the efficiency of crossbreeding, it is necessary to understand the molecular mechanisms of heterosis.

Some methods such as physiological, genetic, molecular, transcriptomic and proteomic approaches, have been used to study heterosis for many years [1, 3, 4]. Among of them, physiological studies revealed that heterosis is affected, to some extent, by the magnitude and rate of vegetative growth, the flowering time, the yield, and the resistance levels to biotic and abiotic environmental stresses [5]. A genetic study showed that one showing primarily nonadditive gene action explained most of the heterotic variation of grain weight in rice [6]. Recently, much attention has been paid to heterosis associated genes [1, 7]. As we known, various genes associated with heterosis have been located or been deeply studied, such as GA-related genes [8], ErbB-3 binding Protein 1 [9], calcium-dependent protein kinase [10, 11], xyloglucan endotransglucosylase/hydrolase [12], cell number regulator gene [13]. With the development of life sciences-related technology, some new methods have also been used to study heterosis, including transcriptomic methods, especially proteomics [1, 4]. Transcriptomics technologies also have been used to compare parental lines with their F1 hybrids to identify genes potentially involved in heterosis, and fond that the mode of gene action in F1 hybrids is classified as having additive and non-additive expression [14]. In proteomics analysis of crop hybrids, some heterosis-related differentially accumulated proteins (DAPs) were identified and non-additive proteins (NAPs) were believed as the important DAPs [15, 16]. In analysis of heterosis-related proteins, proteins that deviated from the midparent value are termed non-additive proteins (NAPs), while others are termed additive proteins (APs). The concept of NAPs is widely used in heterosis research and NAPs are generally classed into four types, ‘above-high parent abundance’ (++), ‘high parent abundance’ (+), ‘below-low parent abundance’ (− −) and ‘low parent abundance’ (−) [16, 17]. Above-high or below-low parent abundance indicates that the NAP content in hybrid is significantly greater or lower than any of parents, high or low parent abundance indicates that the NAP content in hybrid is significantly greater or lower than one of parental line but not another parental line.

Maize is one of the most important cereal crops worldwide and maize hybrids with heterosis are wildly planted in the world [18]. Quantitative trait locus analyses at earlier stage concluded that overdominance is the main cause of heterosis [19], and at recently, multiparent populations have been used to identify quantitative trait locus associated with the genetic architecture of hybrid performances [20, 21]. Transcriptome analyses also have been performed using seedlings, roots, embryos, and immature ears in maize [22, 23]. Calcium-dependent protein kinases are calcium-binding serine/threonine protein kinases, play essential roles in cellular calcium signaling processes. A putative CDPK gene, ZmCPK16 was reported to had different expressions between hybrids and their parents in maize, indicated it plays a part in heterosis [10]. Xyloglucan endotransglucosylase/hydrolase play an essential role in the construction and restructuring of xyloglucan cross-links, a family of xyloglucan modifying enzymes [12], a xyloglucan endotransglucosylase/hydrolase (GRMZM2G091118) down-regulated at V3 stem and shoot apical meristem, although it up-regulated at 6 days after sowing primary root, and had consistent expression at others tissues in different heterosis groups [24]. The cell number regulator super family negative regulators across diverse species. Cell number regulator 2 expression was found to be negatively correlated with tissue growth activity and hybrid seedling vigor in maize [13]. Several heterosis-related genes in maize have been analyzed, but it is not easy to understand the causes or the results of heterosis.

In addition to using nucleic acid research methods, proteomic approaches have successfully identified some maize heterosis-related DAPs in different tissues and/or stages. In earlier research, by using two-dimensional gel electrophoresis (2DE) and ESI-MS/MS, it was found that NAP accumulation in primary roots of maize hybrids might be associated with the manifestation of heterosis [25]. In 2010, 2DE and ESI-MS/MS were also used to study NAP accumulation patterns in maize hybrids during embryo development [15]. An analysis of the heterosis of ZD909 determined that half of the DAPs in immature maize ears were related to dominance expression patterns, and high parental dominance proteins mainly participated in carbon metabolism and nitrogen assimilation processes [26]. In 2018, a proteomics analysis of DAPs in the primary roots of the popcorn hybrid UENF/UEM01 showed that the heterosis was associated with an up-regulation of proteins related to synthesis and energy metabolism at an early stage of plant development [16]. A proteomics study of auxin homeostasis’ influence on the heterosis of the eighth internodal length in maize found that proteins involved in phenylpropanoid and benzoxazinoid metabolic pathways were correlated with the extent of the heterosis [27]. Recently, 2DE and MALDI-TOF-MS were also used to analyze the seed germination trait of hybrid DHM 117 and its parental inbred lines. The DAPs between the hybrid and its parental inbred lines were related to metabolism and energy, followed by storage proteins, stress response, transcription and translation [28]. These results indicated that hybridization between parents can change the expression types of some proteins in maize hybrids, which might lead to heterosis. Understanding the proteomic characteristics of maize hybrids and their parents, and determining the protein types associated with the advantages of hybrids, may provide important guidance for efficient breeding.

‘Zhongdan808’ (ZD808) and ‘Zhongdan909’ (ZD909) are excellent maize hybrids with high yield, good quality, multi-resistance, and wide adaptability. The hybrid ZD808 was bred using CLll as the female parent and NG5 as the male parent, and it is mainly planted in southwestern China. Another hybrid, ZD909, was bred using Z58 as the female parent and HD568 as the male parent, and it is mainly planted in the Huang-Huai-Hai area of China. The two hybrids have been grown on over 6.67 million hectares of cropland in the past in China. They both exhibit strong heterotic phenotypes compared with their parents for agronomic traits, such as more vigorous seedlings, larger ears, bigger grain size, and increased stress resistance. Under water stress conditions, ZD808 had a higher relative water content, lower malondialdehyde content and better osmotic adjustment ability compared with other varieties [29]. However, the mean leaf area index and duration, as well as the mean net assimilation rate, were higher in ZD909 compared with other varieties [30]. In the present study, we used LC/MS/MS to analyze DAPs and NAPs in seeding leaves of hybrids ZD808 and ZD909. This may provide useful information for understanding of the molecular mechanisms involved in the heterosis of hybrid maize.

## Results

**Fig. 1:**
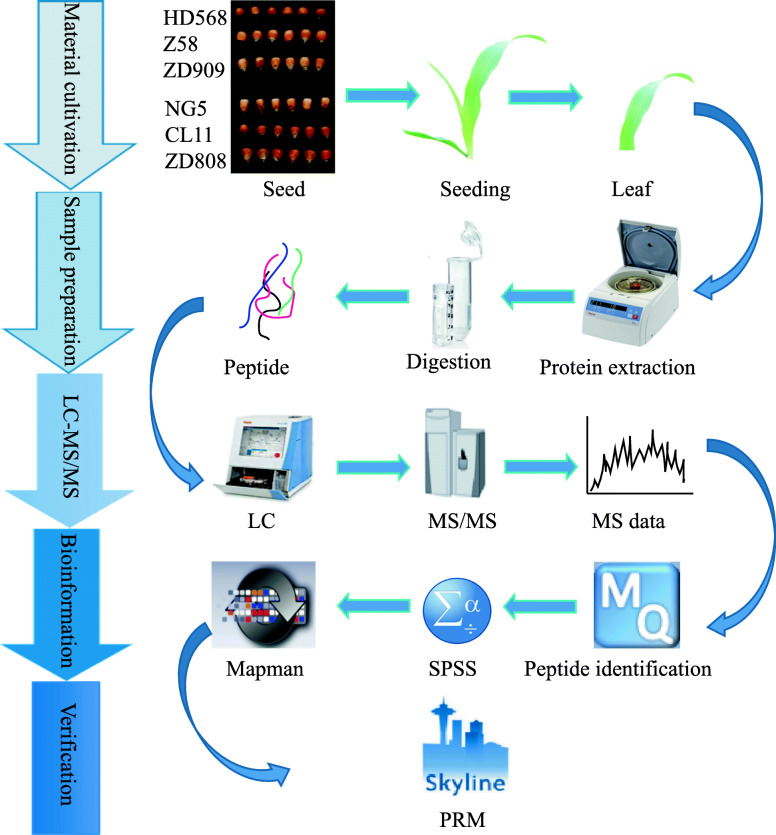
Flow chart of the proteomics analysis of maize hybrids and their parental lines

By using a comparative proteomics approach (Fig. 1), proteomes in seeding leaves of maize hybrids ZD808 and ZD909, and their parents were analyzed and 12 heterosis-related proteins were verified.

### LC-MS analysis and statistical analysis of quantitative identified proteins

**Fig. 2:**
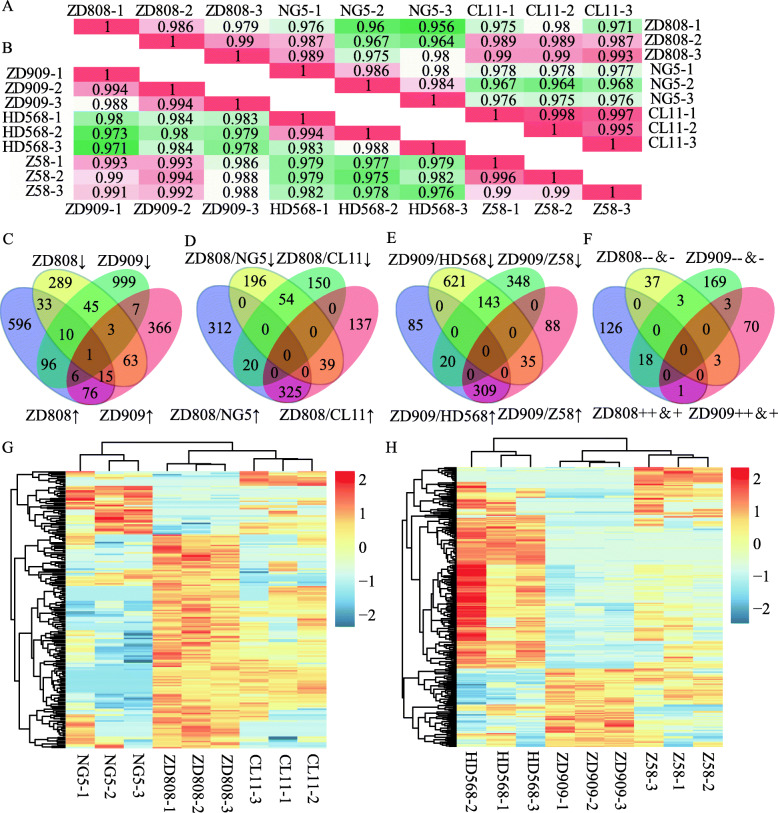
Statistical analysis of three biological repeats of two hybrids and their parental lines. (A) Pearson’s correlation analysis of maize hybrid Zhongdan 808 and its female parent CL11 and male parent NG5. (B) Pearson’s correlation analysis of maize hybrid Zhongdan 909 and its female parent Z58 and male parent HD568. (C) Venn diagram of up-regulated and down-regulated DAPs in hybrids compared independently with the female and male parents. (D) Venn diagram of up-regulated and down-regulated DAPs in Zhongdan 808 when compared with its female parent CL11 and male parent NG5. (E) Venn diagram of up-regulated and down-regulated DAPs in Zhongdan 909 when compared with its female parent Z58 and male parent HD568. (F) NAPs in Zhongdan 808 and Zhongdan 909. (G) Heatmaps for cluster analysis of all the non-additive proteins in Zhongdan 808 and its parents. (H) Heatmaps for cluster analysis of all the non-additive proteins in Zhongdan 909 and its parents. ‘ZD808’ indicates Zhongdan 808, ‘ZD909’ indicates Zhongdan 909, ‘↑’ indicates a protein up-regulated in hybrids by at least a 2-fold change compared with any parent, ‘↓’ indicates a protein down-regulated in hybrids by at least a 2-fold change compared with one parent, ‘+’ indicates high parent abundance NAPs, ‘++’ indicates above-high parent abundance NAPs, ‘−’ indicates low parent abundance NAPs, ‘− −’ indicates below-low parent abundance NAPs. The selection criterion for DAPs was fold change > 2, and the criteria for NAPs was fold change > 1.5 and *p* < 0.05

The total ion chromatograms of those samples showed that the nano liquid phase peaked within 10 min, and the distribution of the chromatographic peaks was uniform and smooth (Fig. S1, S2, S3, S4, S5 and S6: Total ion chromatograms of all the samples). Thus, the mass spectrometry system appeared stable, the parameters were suitable, and the repeatability of the sample preparation was good. Mass spectrometry raw files from three biological repeats of six varieties were used for the quantitative identification through a label-free MS-based qualitative proteomics technique. A total of 2222 and 2486 proteins were quantitatively identified from ZD808 and its parents (Table S1) and from ZD909 and its parents (Table S2), respectively. According to the Pearson’s correlation analysis of the LFQ quantitative data, the correlations between the three independent biological replicates of most varieties were high (Fig. 2a, b). In the ZD808 group, except for the first biological replicate of ZD808, the other two biological replicates had high similarity levels with the female parent and low similarity levels with the male parent. The difference between the ZD808 male and female parents was greater than that between the hybrid and the parents (Fig. 2a). A similar situation was found in the ZD909 sample group (Fig. 2b). Thus, the protein expression characteristics of the two hybrids came more from their female parents than their male parents. In addition, principal component analysis was conducted in all of samples, the PC1 and PC2 represented 94.22 and 2.66% of the total variance, respectively, thus making 96.88% for the total variance accounted for in the principal component analysis model (Fig. S7).

To identify the DAPs among samples, a 2-fold change was chosen as an empirical cutoff. In total, there were 833 up-regulated proteins and 459 down-regulated proteins in hybrid ZD808 when compared with its female parent CL11 or male parent NG5, and there were 537 up-regulated proteins and 1167 down-regulated proteins in hybrid ZD909 when compared with its female parent Z58 or male parent HD568 (Fig. 2c). Among the DAPs, there were 325 common up-regulated proteins and 54 common down-regulated proteins in ZD808 when compared with both parents, and there were 309 common up-regulated proteins and 143 common down-regulated proteins in ZD909 when compared with both parents (Fig. 2d, e). NAPs may be highly associated with heterosis. There were 188 and 263 NAPs identified in the seedlings of hybrids ZD808 and ZD909, respectively. Among them, 65 proteins were classified as ‘++’, 80 as ‘+’, 23 as ‘− −’, and 20 as ‘−’ in ZD808 (Table S3). While in ZD909, 22 proteins were classified as ‘++’, 52 were classified as ‘+’, 81 as ‘− −’, and 108 as ‘−’ (Table S4). Among all the NAPs identified from ZD808 and ZD909, only four were identified at the same time in both hybrids (Fig. 2f). The NAPs in two hybrids were clustered, it can be clearly seen that the distribution of NAPs between two hybrids is difference, and the difference between male and female parents is larger than the difference between hybrid and one of the parents in ZD808, the same to ZD909 (Fig. 2g, h).

A comparison of the DAPs in ZD808 and ZD909 showed that the number of common up-regulated DAPs was greater than common down-regulated DAPs. Among the NAPs, the number of above-high and high parental abundance NAPs was greater than below-low and low parental abundance NAPs in ZD808, but to the opposite was true in ZD909. A comparison of the DAPs and NAPs in ZD808 and ZD909 showed that the number of common up-regulated proteins in ZD808 when compared with both parents was higher than the number of NAPs in ZD808 classified as ‘++’ and ‘+’. This was the same for the common down-regulated proteins in both ZD808 and ZD909.

### Pathway enrichment analysis of DAPs and NAPs

**Fig. 3:**
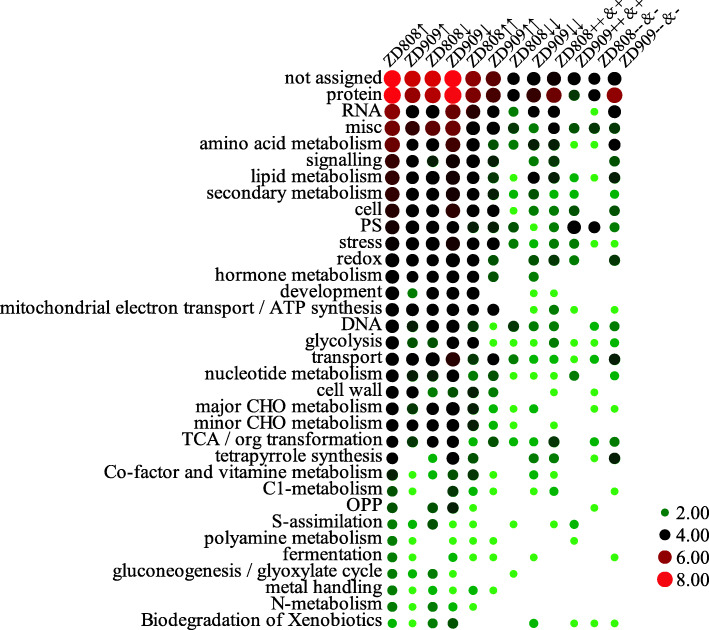
Pathway classification of DAPs and NAPs identified from Zhongdan 808 and Zhongdan 909. MapMan software was used to conduct the functional categorization. The numbers of DAPs and NAPs enriched in pathways were subjected to log2 transformation. Each size and color of circles indicated the number of DAPs or NAPs in hybrids when compared with its parents

The pathways of DAPs and NAPs identified from ZD808 and ZD909 were enriched using MapMan software and divided into 34 classes (Fig. 3). DAPs with at least 2-fold change in the hybrids when compared to any parent, were highly enriched in protein, RNA, amino acid metabolism, signaling, lipid metabolism, secondary metabolism, Photosynthesis (PS), stress, redox, hormone metabolism, and development. up-regulated DAPs with at least 2-fold change in the hybrids when compared to both parents were commonly enriched in many pathways, except development and tetrapyrrole synthesis pathways, which were only enriched in ZD808. down-regulated DAPs with at least 2-fold change in the hybrids when compared to both parents were associated with protein pathways, and in ZD909 were especially enriched in secondary metabolism. NAPs of ZD808 classified as ‘++’ and ‘+’ were associated with protein, RNA, miscellaneous, secondary metabolism, amino acid metabolism, redox, signaling, and tricarboxylic acid (TCA)/org transformation pathways, while NAPs of ZD909 classified as ‘++’ and ‘+’ were mainly associated with the PS pathway. NAPs of ZD808 classified as ‘− −‘and ‘−’ were associated with protein, PS, and miscellaneous pathways. NAPs of ZD909 classified as ‘− −‘and ‘−’ were associated with protein, RNA, amino acid metabolism, transport, secondary metabolism, tetrapyrrole synthesis, miscellaneous, and redox pathways.

### Stress related pathways enhanced in ZD808

**Fig. 4:**
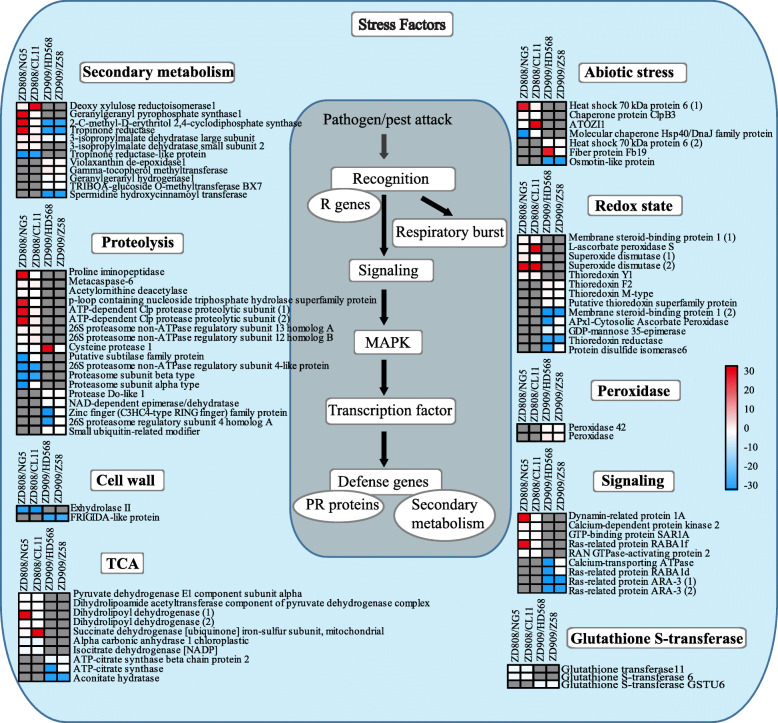
Heat map of NAPs involved in stress factors. The NAPs grouped into stress were visualized using MapMan software. Each square and color indicated the fold change of a NAP in hybrids when compared with the parents. Blue and red indicate a decrease and increase, respectively, in fold change compared with the parents, and gray indicates unidentified. MAPK, mitogen-activated protein kinase; PR, pathogenesis-related

A more detailed analysis showed that a total of 76 NAPs from ZD808 and ZD909 could be enriched in stress response-related pathways (Fig. 4), including secondary metabolism, proteolysis, TCA, abiotic stress, redox state, signaling and so on. Among them, 44 NAPs were identified from ZD808 and its parents, and 32 NAPs were up-regulated in ZD808. At the same time, 36 NAPs were identified from ZD909 and its parents, and only 11 NAPs were up-regulated in ZD909.

### PS related pathways enhanced in ZD909

**Fig. 5:**
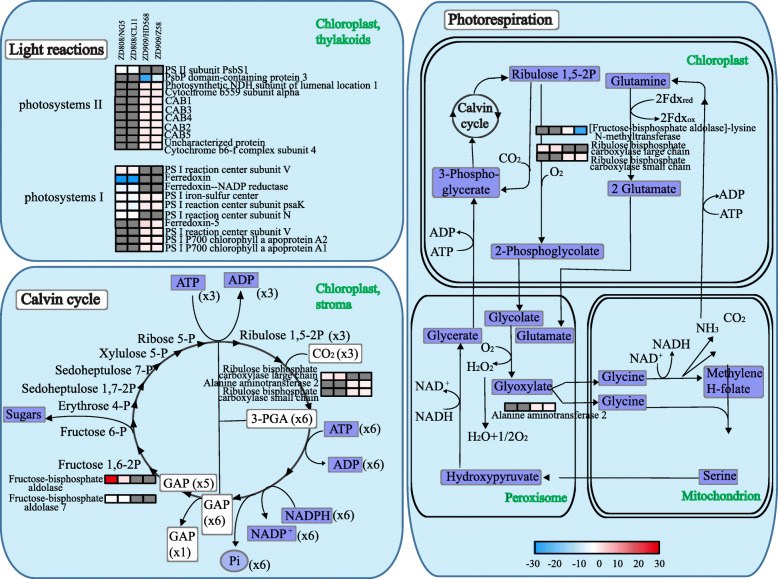
Heat map of NAPs involved in photosynthesis. The NAPs grouped into photosynthesis were visualized using MapMan software. Each square and color indicated the fold change of a NAP in hybrids when compared with the parents. Blue and red indicate a decrease and increase, respectively, in fold change compared with the parents, and gray indicates unidentified. Chlorophyll a-b binding protein (tr|B6SSN3|B6SSN3_MAIZE, CAB1; tr|B4FL55|B4FL55_MAIZE, CAB2; tr|B6SUC4|B6SUC4_MAIZE, CAB3; tr|K7TXI5|K7TXI5_MAIZE, CAB4; tr|B6SZT9|B6SZT9_MAIZE, CAB5)

A total of 27 NAPs from ZD808 and ZD909 could be enriched in PS-related pathways (Fig. 5), including light reactions, Calvin cycle and photorespiration. Most of PS-related NAPs were identified from ZD909 and its parents, and many of them were up-regulated, except 2 NAPs were down-regulated in ZD909, they were mainly involved in light reactions. Ten of them were identified from ZD808 and its parents, and only 3 NAPs were up-regulated in ZD808, them were mainly enriched in light reactions and Calvin cycle. The result showed that half of the NAPs of ZD808 involved in PS were classified as ‘− −’ or ‘−’, and unlike in ZD808, most of the NAPs related to PS in ZD909 were classified as ‘+’ or ‘++’.

### PRM verification

**Fig. 6:**
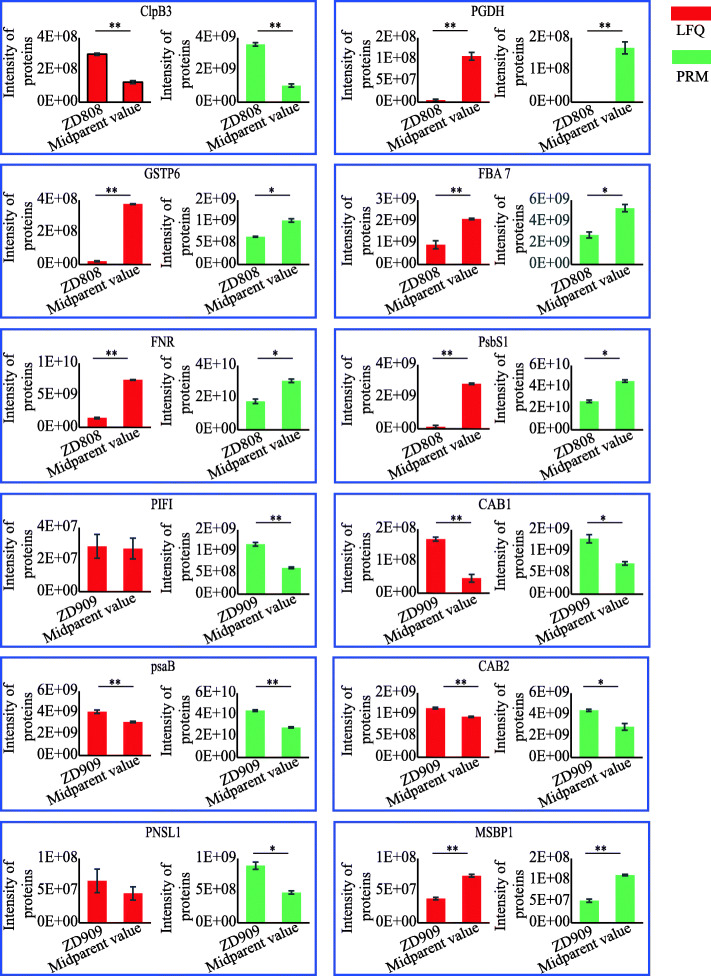
PRM verification of some proteins related to photosynthesis and stress. Stress response-related proteins: chaperone protein ClpB3 (ClpB3), D-3-phosphoglycerate dehydrogenase (PGDH), glutathione S-transferase 6 (GST6), and membrane steroid-binding protein 1 (MSBP1). Photosynthesis-related proteins: fructose-bisphosphate aldolase 7 (FBA7), ferredoxin-NADP reductase (FNR), Photosystem II subunit PsbS1 (PsbS1), post-illumination chlorophyll fluorescence increase (PIFI), chlorophyll a-b binding proteins (CAB), Photosystem I P700 chlorophyll a apoprotein A2 (psaB), and photosynthetic NDH subunit of lumenal location 1 (PNSL1). ‘Midparent value’ of LFQ data indicated the average LFQ intensity of female and male parents, ‘Midparent value’ of PRM data indicated the average area of female and male parents. ‘**’ indicated *p*-value< 0.01; ‘*‘indicated p-value< 0.05

The PRM assay was also used to verify a subset of the NAPs obtained in the label-free analysis. Because the signature peptides for target proteins must exhibit uniqueness, only proteins that possessed unique peptide sequences were selected for the PRM analysis. Some proteins related to the PS and stress pathways were selected for PRM verification (Fig. 6). The Skyline analysis results of unique peptides of 12 proteins verified by PRM are shown in supplementary files (Fig.S8, S9, Table S7), and the detail data results including peak area and retention time of each peptide fragment and the peptide information are shown in supplementary files (Table S5, S6). The verification results showed that the intensity of most PS-related proteins in hybrid ZD808 were lower than in its parents, while the opposite was true in ZD909.

## Discussion

Heterosis is ubiquitous and has attracted much scientific attention. The mechanism of heterosis is very complex. Many methods, like genomics and transcriptomics, have been used in heterosis studies [2], and now proteomics is also being used. ZD808 is the main cultivated variety in southwestern China, which has a complicated eco-environment with barren soil and frequent drought and disease occurrences, while ZD909 is the main cultivated variety in the Huang-Hui-Hai area of China. ZD808 can adapt to adverse environmental conditions and exhibits excellent resistance levels to stresses, such as drought [29], while ZD909 has a strong photosynthetic capacity [30]. So that maize hybrids ZD808 and ZD909 have excellent yield traits compared with those of their parents [26, 29] (Table 1). Here, proteomic techniques were used to analyze the two hybrids and their parental lines to provide new clues regarding the heterosis mechanisms. However, further exploration is needed in the aspects of the breadth, proteomics technology and gene function verification.

**Table 1:** Yield traits of maize hybrids and their parental lines

	Ear length (cm)	Ear diameter (cm)	Kernels row number	Kernels number per row	Hundred-grain weight (g)
ZD909	20.2(±2.464)	5.1(±0.799)	15.3(±0.526)	42.3(±0.755)	37.7(±0.535)
Z58 (Female)	14.9(±2.567) **	4.1(±0.198) **	11.9(±0.342) **	22.6(±0.830) **	33.7(±1.318) *
HD568 (Male)	10.1(±3.423) **	3.9(±0.969) **	14.5(±0.854) **	18.1(±0.670) **	24.7(±0.740) **
ZD808	26.1(±0.515)	5.6(±0.305)	16.0(±0.000)	51.2(±3.633)	36.1(±1.246)
CL11 (Female)	15.1(±0.265) **	4.1(±1.708) **	12.5(±1.000) **	21.0(±1.155) **	24.6(±0.928) **
NG5 (Male)	19.8(±1.301) **	4.7(±1.182) **	16.0(±0.000)	31.8(±1.789) **	25.4(±0.950) **

Note: ‘**’ indicated p-value< 0.01; ‘*’ indicated p-value< 0.05

Heterosis is affected, to some extent, by many factors [3, 5], and NAPs may be a major source of heterosis. By using LFQ, 2222 and 2486 proteins were quantified from seedling leaves of ZD808 group and ZD909 group, respectively, means that there is a difference in the analysis results between groups. The Pearson’s correlation analysis revealed that the correlations of the quantitative proteins of three independent biological replicates were relatively high, and the two hybrids were more similar to their female parents. In our experiment, there has some difference exist in the proteome analysis results of ZD808–1 and the other two biological repeat samples. However, all the data of ZD808 biological repeats were more similar to the female parent but not to the male parent. The results showed that the correlation pattern of ZD808–1 and the other two biological repeats were similar (Fig. 2a, b). Principal component analysis also showed that correlation pattern of two hybrids were more similar to their female parents, and the correlations of ZD909 group was higher than ZD808 group (Fig. S7). Venn diagrams independently illustrated the relationships of DAPs and NAPs between two hybrids. Some DAPs of ZD808 and ZD909 were simultaneously identified as up-regulated proteins and/or down-regulated proteins when compared with any parent, but there were no significant differences between the numbers of simultaneously identified DAPs in most intersections of the two data sets (Fig. 2c). Although many DAPs in ZD808 were up-regulated and DAPs in ZD909 were down-regulated, the comparative analysis of common DAPs indicated that there were more common up-regulated DAPs than common down-regulated DAPs in both ZD808 and ZD909 (Fig. 2d, e). However, the NAP analysis showed that their patterns were significantly different in ZD808 and ZD909 (Fig. 2f). The Cluster analysis showed a difference distribution of NAPs between two hybrids, but similarity of two hybrids and their female parents is higher than hybrids and their male parents, it indicated female parent had more influence on hybrids NAPs.

All the identified proteins were classified into 33 pathways as well as a group in which proteins were not annotated (Fig. 3). The pathway enrichment analysis showed that most of the up-regulated DAPs in ZD808 and down-regulated DAPs in ZD909, when compared with any parent, were enriched in many pathways. In ZD808, DAPs up-regulated by at least 2-fold change in the hybrids when compared with both parents were enriched in almost all of the pathways, especially stress-related pathways such as amino acid metabolism, signaling, lipid metabolism, secondary metabolism, stress, redox, and hormone metabolism. In ZD909, DAPs up-regulated by at least 2-fold change in the hybrids when compared with both parents were mainly enriched in cell, stress, and mitochondrial electron transport/ATP synthesis and transport. Thus, the functions of the DAPs in ZD808 and ZD909 appeared to be different. However, in ZD808, most of the ‘++’ and ‘+’ NAPs were only enriched in stress-related pathways, while ‘− −’ and ‘−’ NAPs were only obviously enriched in PS pathways. However, in ZD909, although ‘− −’ and ‘−’ NAPs were a large majority of all the NAPs, the ‘++’ and ‘+’ NAPs were highly enriched in PS pathway, while ‘− −’ and ‘−’ NAPs were enrichment in RNA, amino acid metabolism, secondary metabolism, and redox. Thus, stress-related pathways were enhanced in ZD808, and PS-related pathways were enhanced in ZD909.

Because non-additively expressed proteins were predicted to play important roles in heterosis [3, 16, 31], all the identified NAPs were further analyzed using MapMan (Table S3, S4). Most of the NAPs identified from the two hybrids could be divided into four classes of pathways, stress responses, PS, amino acid metabolism and protein. We believe that if the majority of the NAPs in a pathway were up- or down-regulated, then the pathway was enhanced or impeded, respectively. Stress responses play crucial roles when plants are subjected to abiotic or biotic stresses. The MapMan analysis showed that some up-regulated NAPs of ZD808 were enriched in pathways involved in stress responses (Fig. 4). Among these proteins, 70-kDa heat shock proteins (Hsp70s) are a widely expressed family with similar structures that exist in almost all organisms. Hsp70s are important components of the cell’s machinery for protein folding, and they help to protect cells from cold, heat, and drought stresses [32–34]. Other proteins shown in Fig. 4 are also related to basal metabolism and stress responses. The basal metabolism and stress responses were enhanced in hybrid ZD808 compared with its parents. Some studies reported the relationship between heterosis and stress tolerance, but a great difference exist on different species and different stress [1]. Some evidence showed that there is negative relationship between expression of defense response genes and growth heterosis [35], there are also some evidences of a positive or no relationship between heterosis and stress tolerance [36, 37]. In this study, stress responses were enhanced at three-leaf stage, and yield increased at maturity stage, it suggested that there had a positive relationship between heterosis and anti-stress in ZD808. Increased vigor or yield is usually the focus of heterosis research. PS is a key process in promoting plant growth and development. The main PS-related pathways enriched with NAPs of ZD808 and ZD909 are shown in Fig. 5. There is a great difference in the PS activity between the two hybrids at the three-leaf stage. The enhanced of PS-related pathways at three-leaf stage could be the bases on heterosis of yield at maturity stage.

Amino acid metabolism also plays important roles in the growth processes of plants. A total of 14 NAPs in ZD808 and ZD909 were enriched in amino acid metabolism-related pathways. Among them, most proteins identified as ‘++’ or ‘+’ in ZD808, except D-3-phosphoglycerate dehydrogenase. However, in ZD909, all of these NAPs were classified as ‘− −’ or ‘−’. It indicated that amino acid metabolism was enhanced in hybrid ZD808 and impeded in ZD909.

Moreover, protein pathways were greatly affected by hybridization, including amino acid activation, protein degradation (serine protease and ubiquitin), protein folding, protein synthesis (elongation, initiation and ribosomal protein), protein targeting (in the chloroplast and nucleus), assembly and cofactor ligation, and posttranslational modification. In ZD808, most of the NAPs related to protein synthesis, folding, amino acid activation, and targeting were classified as ‘+’ or ‘++’. However, in ZD909, most of the NAPs in protein-related pathways were classified as ‘− −’ or ‘−’. This indicated that the effects of protein synthesis and other protein-related pathways in the two hybrids were very different.

In the PRM verification of some key proteins, the abundance levels of 12 NAPs related to PS and stress were analyzed (Fig. 6, Table S7). The PRM results in parents and hybrids were similar to the label-free quantitative proteomics results. Most of them had significant differences between hybrid and midparent value both in LFQ data and PRM verification.

Four stress response-related proteins, chaperone protein ClpB3, D-3-phosphoglycerate dehydrogenase (PGDH), glutathione S-transferase 6 (GST6), and membrane steroid-binding protein 1 (MSBP1), were verified by PRM. The direct interaction of ClpB3 with Hsp70 may eventually result in the refolding, and hence the reactivation, of deoxyxylulose 5-phosphate synthase, which promotes the repair of the methylerythritol 4-phosphate pathway [38–40]. This pathway synthesizes the metabolic precursors of isoprenoids, such as carotenoids, and the prenyl chains of chlorophylls, tocopherols, or plastoquinone. PRM verification classified ClpB3 as ‘++’ in ZD808, while it was classified as ‘+’ in label-free quantitative proteomics, there had significant difference between ZD808 and midparent value in PRM verification. The high abundance level of ClpB3 in hybrid ZD808 indicates that its stress-response capability is greater than that of its parents. PGDH, which originates from glycolysis and the Calvin cycle, participates in the biosynthesis of L-serine [41]. *Arabidopsis thaliana* PGDH1 is also essential for plant adaptation to high CO_2_, as well as for embryogenesis and pollen development [42]. Recently, a study showed that PGDH activity increased in sugar beet after exposure to salt stress [43]. Even though PGDH was down-regulated in ZD808, the response to salt stress-related PGDH in ZD808 may be greater than the responses of its parents. GST is a multifunctional enzyme that participates in reducing the formation of reactive oxygen species and cell toxicity [44, 45]. GST6 is up-regulated under salt-stress conditions [46]. GST6 was classified as a ‘−’ NAP in ZD808, and a significant difference exist in ZD808 and its midparent value in PRM verification. Thus, the response to salt stress-related GST6 may be high in ZD808. MSBP1 inhibits cell elongation [47] and negatively regulates brassinosteroid signaling [48]. MSBP1 expression is also closely related to salinity tolerance [49]. Like many stress-related NAPs in ZD909, this protein was down-regulated, which was verified by PRM.

Eight proteins related to PS, fructose-bisphosphate aldolase 7 (FBA7), ferredoxin-NADP reductase (FNR), photosystem II subunit PsbS1 (PsbS1), post-illumination chlorophyll fluorescence increase (PIFI), chlorophyll a-b binding proteins (CAB1 and CAB2), photosystem I P700 chlorophyll a apoprotein A2 (psaB), photosynthetic NDH subunit of lumenal location 1 (PNSL1), were also subjected to PRM verification (Fig. 6). Except PIFI and PNSL1, the other six proteins related to PS were verified by PRM. These proteins had important roles in PS. PIFI, CAB1 and 2, psaB, and PNSL1 were identified in ZD909 as ‘+’ NAPs, and most of them were classified as ‘+‘or ‘++’ in the PRM verification. PIFI is essential for NDH-mediated nonphotochemical reduction of the plastoquinone pool in chlororespiratory electron transport [50]. CABs are a class of important proteins in thylakoid membranes of higher plants, and take part in both photosystems I and II. In addition, CABs may be involved in abiotic stress responses [51]. It was also found that CABs were highly expressed in the F1 hybrid of Chinese cabbage at two days after sowing, means that early developmental events in the germinating seedling of the hybrid may be important for later developmental vigor and yield advantage [52]. PsaB a primary electron donor of photosystem I. PNSL1 is a lumen subcomplex L subunit of chloroplast NDH. Unlike in ZD909, most proteins related to PS identified by label-free quantitative proteomics were down-regulated, and FBA7, FNR, and PsbS1 classifications were verified by the PRM. FBAs participate in carbon fixation, glycolysis, and Calvin cycle pathways. FNR catalyzes reversible electron transport between ferredoxin and NADP. PsbS1 is a subunit of photosystem II. These roles indicated that PS was decreased in ZD808 compared with its parents. Based on the label-free quantitative proteomics and PRM analyses of NAPs, the heterosis of maize hybrids ZD808 and ZD909 may be mainly related to PS and/or stress resistance.

In addition to the above-mentioned proteins which related to stress responses and photosynthesis, a protein expressed one of reported genes associated with heterosis, ERBB-3 binding protein 1 (EBP1), was up-regulated in ZD909 (Table S2). EBP1 regulates organ size through cell growth and proliferation [9]. And more, some DAPs/NAPs in ZD808 and ZD909 are not yet functionally annotated (Table S1, S2). And even though analysis of DAPs and NAPs in the germinating seedling of hybrids ZD808 and ZD909 has given us some useful information in understanding the reason of valuable agronomic traits, proteomics research throughout the growth stage is still lacking. All these results indicate that the mechanism of maize heterosis needs further study.

## Conclusions

In summary, nearly 200 NAPs were identified from seedling leaves of maize hybrids ZD808 and ZD909, and most of them were enriched in stress responses, PS, amino acid metabolism and protein pathways. Pathways related to stress response, proteins, and amino acid metabolism were significantly enhanced in hybrid ZD808 but impeded in ZD909. However, pathways related to PS were significantly enhanced in hybrid ZD909. The results show that the biological basis of heterosis in the two maize hybrids is different. In comparison with their parents, the excellent agronomic traits of hybrid ZD808 was correlated with the high expression levels of some proteins related to stress responses and metabolic functions, while those of ZD909 was correlated with photosynthesis. Our proteomics results support previous physiological and morphological research and have provided useful information in understanding the reason of valuable agronomic traits.

## Methods

### Plant materials and growth conditions

The seeds of maize hybrid ZD808 (NG5 × CL11), along with its female parent CL11 (from 78,599) and male parent NG5 (from 95,236 × 95,167, Iowa State University), and those of the hybrid ZD909 (Z58 × HD568), along with its female parent Z58 and male parent HD568 (from DKC79 × Chang7–2) were acquired from professor Changling Huang’s lab (Center for crop genetics and breeding, Institute of crop science, Chinese Academy of Agricultural Sciences) in this study and some yield traits data are shown in Table 1. The seeds of each variety were washed separately with distilled water, soaked in 2% NaClO for 20 min, and washed again with distilled water. Then, ~ 200 seeds were germinated and cultured in sand for 15 days with a 16 h light at 28 °C, and 8 h dark at 24 °C photocycle and 70% relative humidity. Then, the seedlings with uniform growth were transplanted into new sand supplemented with 1/2 nutrient solution. Finally, the second leaves were collected at three-leaf stage, frozen in liquid nitrogen, and then stored at − 80 °C for the proteome analysis.

### Protein extraction and digestion

Proteins were extracted according to Zhu et al. [53]. Maize leaves of each sample (100 mg, three biological repeats and three technical repetitions) were used for protein extraction. The extracted proteins were digested by 50 μL trypsin and incubated overnight at 37 °C, centrifuged at 13,000×*g* for 30 min and stored at − 20 °C until further use.

### Mass spectrometric analysis

Polypeptide fragments were separated and analyzed using an Easy nLC 1000 coupled to a Q-Exactive Plus (Thermo Fisher Scientific). Parameter setting of mass spectrometer were the same to Zhu et al. [53]. The experiments with each sample were repeated at least three times, and then 54 mass spectrometry raw files with high reliability were obtained (Supplementary data: 54 mass spectrometry raw files, Partner repository with the dataset identifier PXD015819.).

### Protein identification and LFQ

The label-free quantitative (LFQ) of proteins was carried out using MaxQuant software (version 1.6.0, https://www.coxdocs.org) and based on the Uniprot database (Maize, August 28, 2018). The mass tolerance of the precursor was set as 20 ppm, 0.02 Da fragment ion tolerance; up to two missed cleavages, carbamidomethyl cysteine as a fixed modification, N-terminal acetylation and methionine oxidation of proteins were defined as variable modifications. The cutoff global false discovery rate (FDR) for peptide and protein identification was set at 0.01.

### Bioinformatics analyses

The LFQ data were preprocessed using a standardized method before use in screening related proteins. A Pearson’s correlation analysis was used to evaluate the quality of the original data (SPSS 22.0, https://www.ibm.com/analytics). A Venn analysis (https://bioinfogp.cnb.csic.es/tools/venny/) was used to group the DAPs and NAPs between hybrids and their parents. Based on previous literature, the selection criterion for the DAPs was fold change > 2, and the criterion for the NAPs was fold change > 1.5 and *p* < 0.05. Principal component analysis was visualized using R package ggplot2_3.3.0 and ggfortify_0.4.9. Heatmap was visualized using R package pheatmap_1.0.12, and scaled row using Z-score. Finally, pathway enrichment analyses of DAPs and NAPs were performed using MapMan (https://MapMan.gabipd.org/).

### Parallel reaction monitoring (PRM)

A PRM analysis were performed on an Easy nLC 1000 coupled to a Q-Exactive Plus (Thermo Fisher Scientific). Leaves were collected at three-leaf stage. Proteins were extracted and digested, and then used for PRM analysis. The mass spectrometry parameters included an isolation width of ± 1 Thomson/window of 2 Thomson for target precursor ions, and precursor ions were fragmented through higher energy collisional dissociation with normalized collision energies of 27 eV. In all the experiments, a full mass spectrum at 70,000 resolution relatives to *m/z* 200 was followed by up to 30 PRM scans at a 17,500 resolution. The other separation conditions and mass spectrometry parameters were as described above. Finally, raw data obtained were analyzed using Proteome Discoverer 2.1 (Thermo Electron, Germany) and Skyline 4.1 software (downloaded from ftp://ftp.ensembl.org/pub/mnt2/).

## Supplementary Information

**Additional file 1 Fig. S1.** Total ion chromatograms of ZD808.

**Additional file 2 Fig. S2.** Total ion chromatograms of ZD808 female parent.

**Additional file 3 Fig. S3.** Total ion chromatograms of ZD808 male parent.

**Additional file 4 Fig. S4.** Total ion chromatograms of ZD909.

**Additional file 5 Fig. S5.** Total ion chromatograms of ZD909 female parent.

**Additional file 6 Fig. S6.** Total ion chromatograms of ZD909 male parent.

**Additional file 7 Fig. S7.** Principal component analysis of six samples.

**Additional file 8 Fig. S8.** Skyline analysis results of 6 PRM verified proteins unique peptides in ZD808.

**Additional file 9 Fig. S9.** Skyline analysis results of 6 PRM verified proteins unique peptides in ZD909.

**Additional file 10 Table S1.** Quality detection of mass spectrometry of ZD808 and its parents.

**Additional file 11 Table S2.** Quality detection of mass spectrometry of ZD909 and its parents.

**Additional file 12 Table S3.** Details of non-additive proteins of ZD808.

**Additional file 13 Table S4.** Details of non-additive proteins of ZD909.

**Additional file 14 Table S5.** Details of PRM verification data.

**Additional file 15 Table S6.** Peptide information of PRM verification.

**Additional file 16 Table S7.** Details of 12 heterosis-related proteins.

## Data Availability

The mass spectrometry proteomics data have been deposited to the ProteomeXchange Consortium (http://proteomecentral.proteomexchange. org) via the iProX partner repository with the dataset identifier PXD015819 (http://proteomecentral.proteomexchange.org/cgi/GetDataset?ID=PXD015819).

## Abbreviations

NAP: non-additive protein
DAP: differentially accumulated protein
ZD808: Zhongdan808
ZD909: Zhongdan909
LFQ: label-free quantification
AGC: automatic gain control
PRM: parallel reaction monitoring
LC-MS/MS: Liquid chromatography-tandem mass spectrometry
2DE: two-dimensional gel electrophoresis
MALDI-TOF-MS: Matrix-Assisted Laser Desorption/ Ionization Time of Flight Mass Spectrometry
PS: Photosynthesis
TCA: tricarboxylic acid
MAPK: mitogen-activated protein kinase
PR: pathogenesis-related
ClpB3: chaperone protein ClpB3
PGDH: D- 3-phosphoglycerate dehydrogenase
GST6: glutathione S-transferase 6
MSBP1: membrane steroid-binding protein 1
FBA: fructose-bisphosphate aldolase
FNR: ferredoxin-NADP reductase
PsbS1: Photosystem II subunit PsbS1
PIFI: post-illumination chlorophyll fluorescence increase
CAB: chlorophyll a-b binding protein1
psaB: Photosystem I P700 chlorophyll a apoprotein A2
PNSL1: photosynthetic NDH subunit of lumenal location 1
