# Supplementary figures and images for: Comparative proteomic analysis reveals that the Heterosis of two maize hybrids is related to enhancement of stress response and photosynthesis respectively
Source: BMC Plant Biol. 2021 Jan 9;21:34. doi: 10.1186/s12870-020-02806-5 (PMC7796551; doi:10.1186/s12870-020-02806-5)

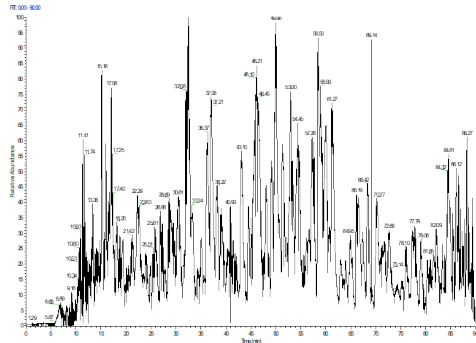

Supplement: Supplementary file 1 — Additional file 1 Fig. S1. Total ion chromatograms of ZD808. [file 12870_2020_2806_MOESM1_ESM.pdf]

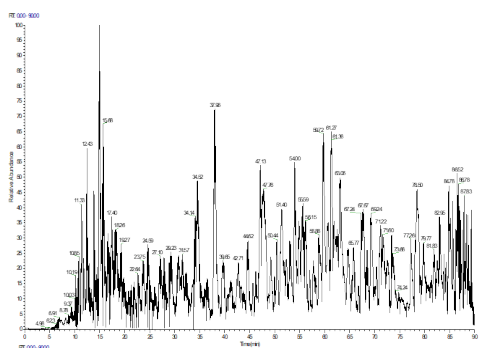

Supplement: Supplementary file 2 — Additional file 2 Fig. S2. Total ion chromatograms of ZD808 female parent. [file 12870_2020_2806_MOESM2_ESM.pdf]

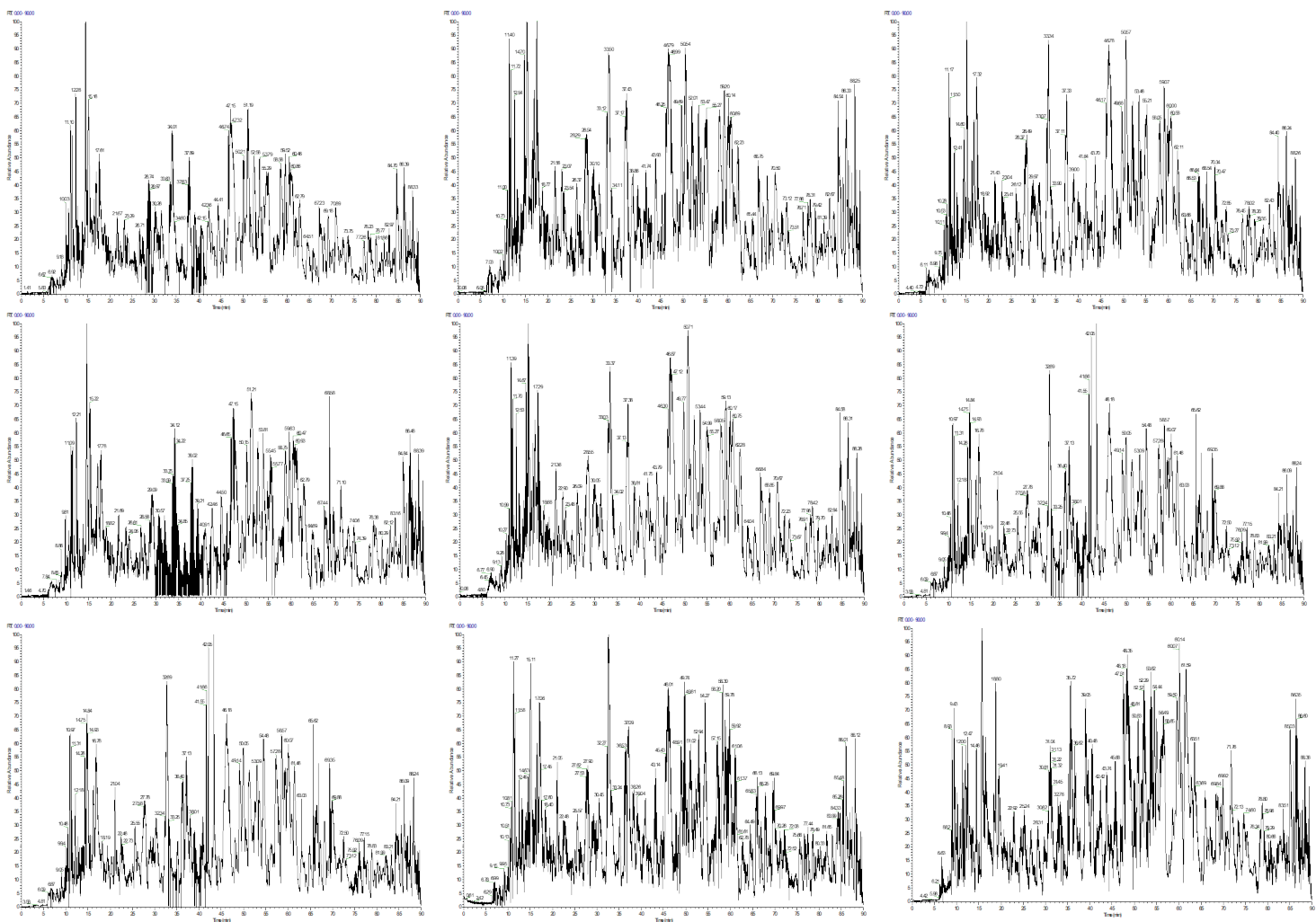

Supplement: Supplementary file 3 — Additional file 3 Fig. S3. Total ion chromatograms of ZD808 male parent. [file 12870_2020_2806_MOESM3_ESM.pdf]

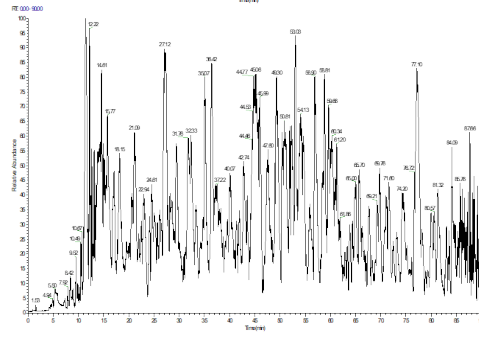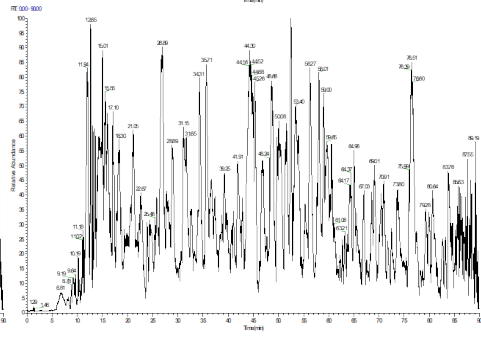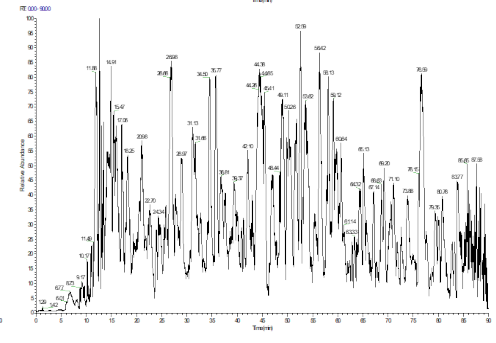

Supplement: Supplementary file 4 — Additional file 4 Fig. S4. Total ion chromatograms of ZD909. [file 12870_2020_2806_MOESM4_ESM.pdf]

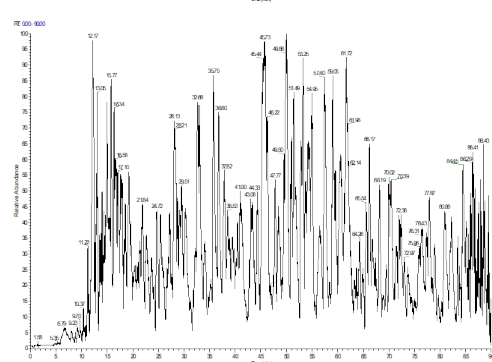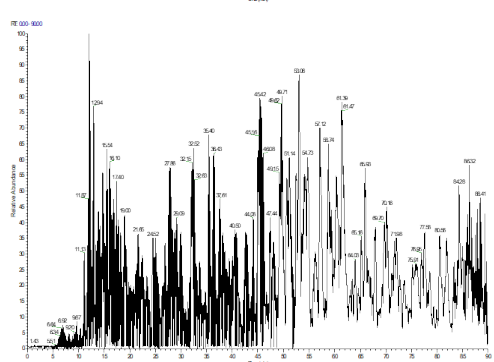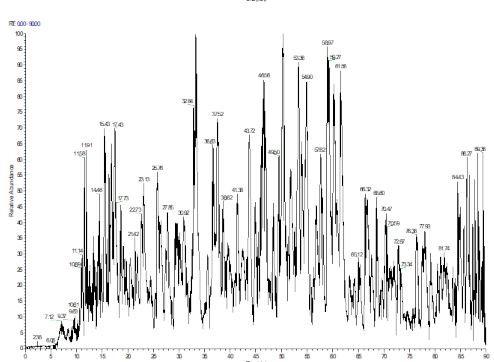

Supplement: Supplementary file 5 — Additional file 5 Fig. S5. Total ion chromatograms of ZD909 female parent. [file 12870_2020_2806_MOESM5_ESM.pdf]

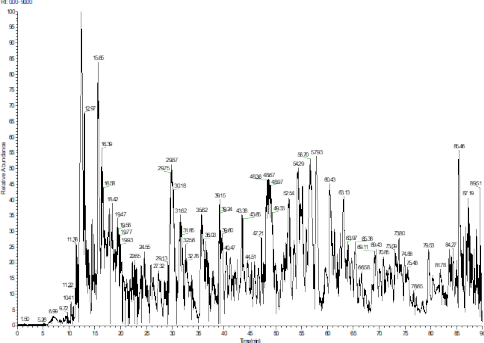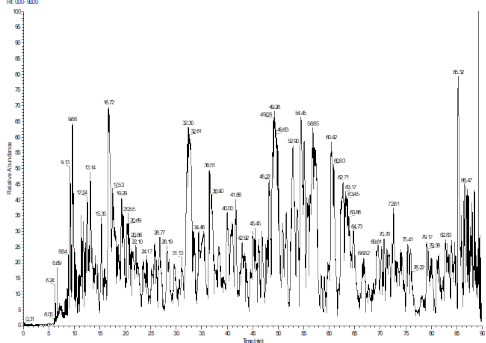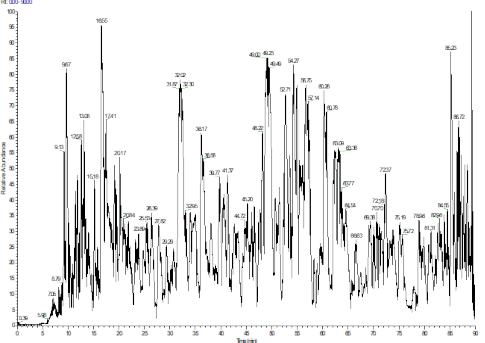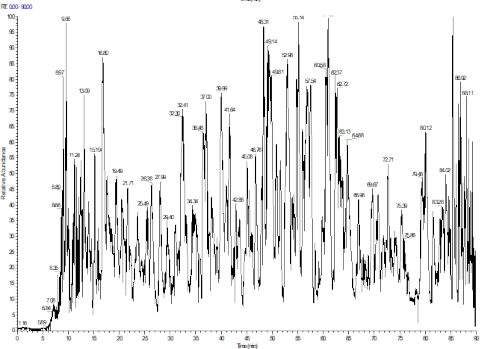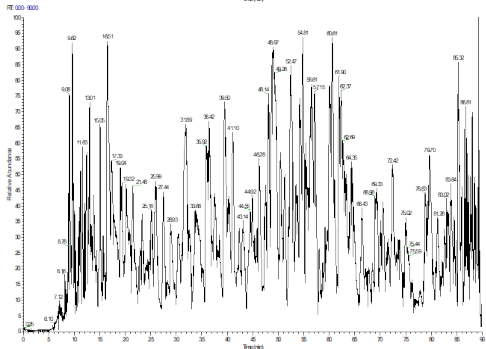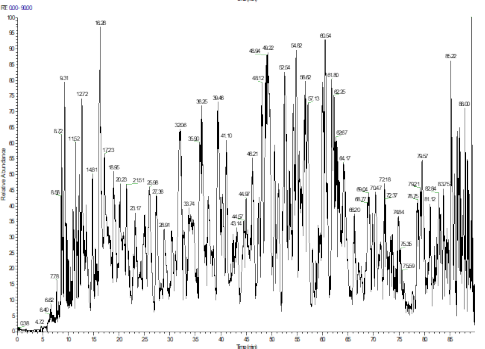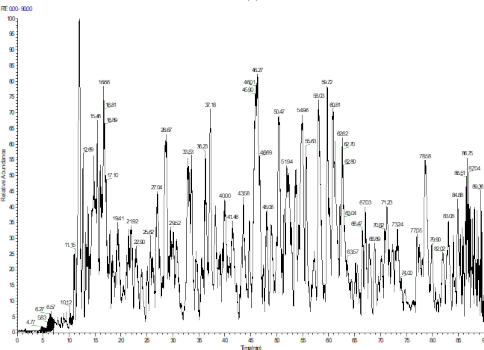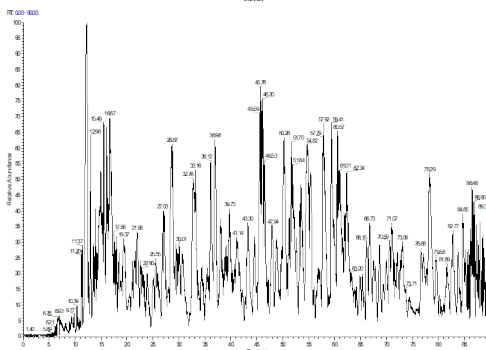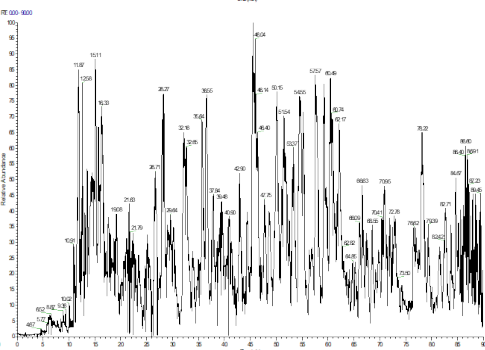

Supplement: Supplementary file 6 — Additional file 6 Fig. S6. Total ion chromatograms of ZD909 male parent. [file 12870_2020_2806_MOESM6_ESM.pdf]

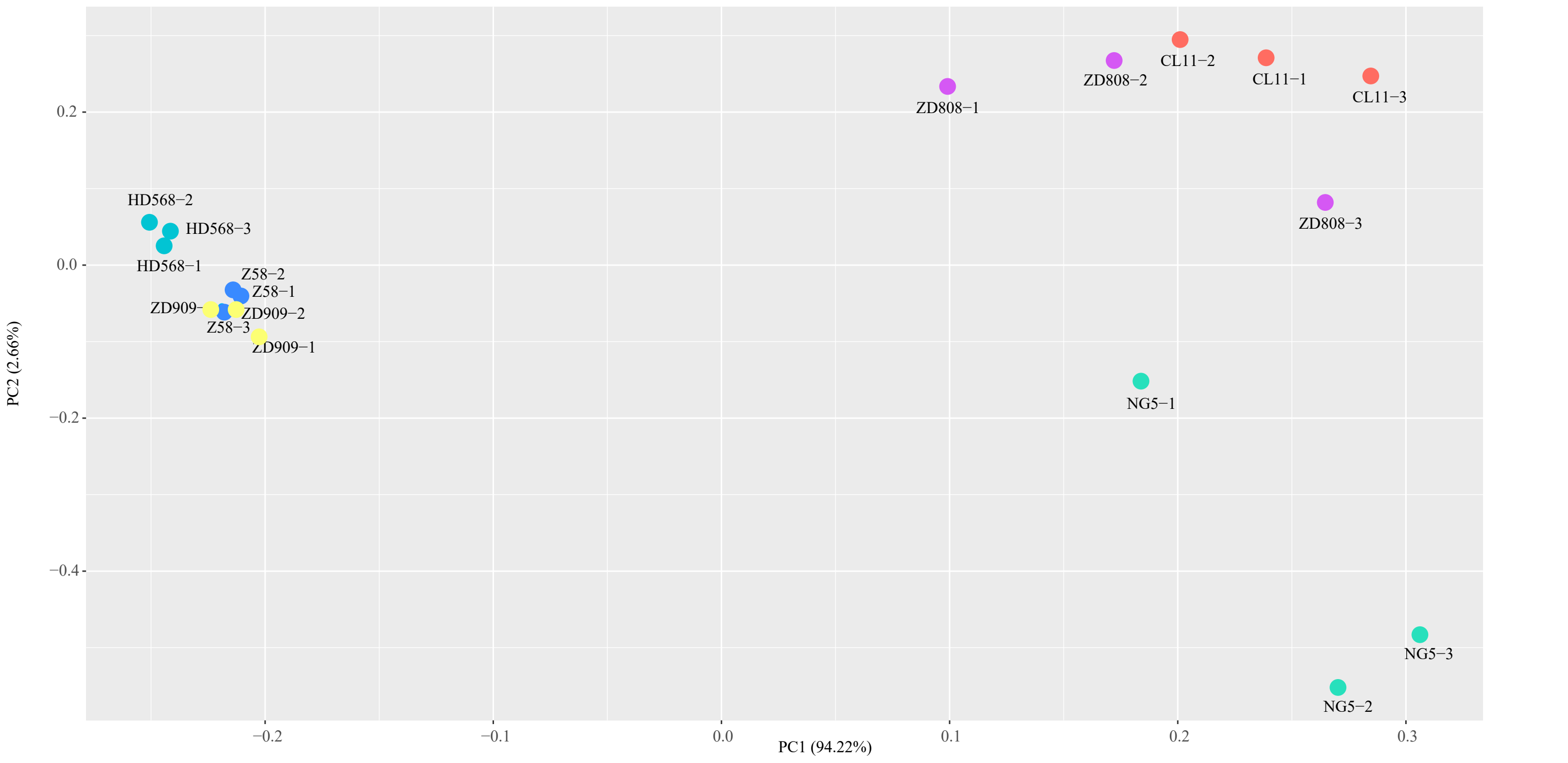

Supplement: Supplementary file 7 — Additional file 7 Fig. S7. Principal component analysis of six samples. [file 12870_2020_2806_MOESM7_ESM.pdf]

PIFI

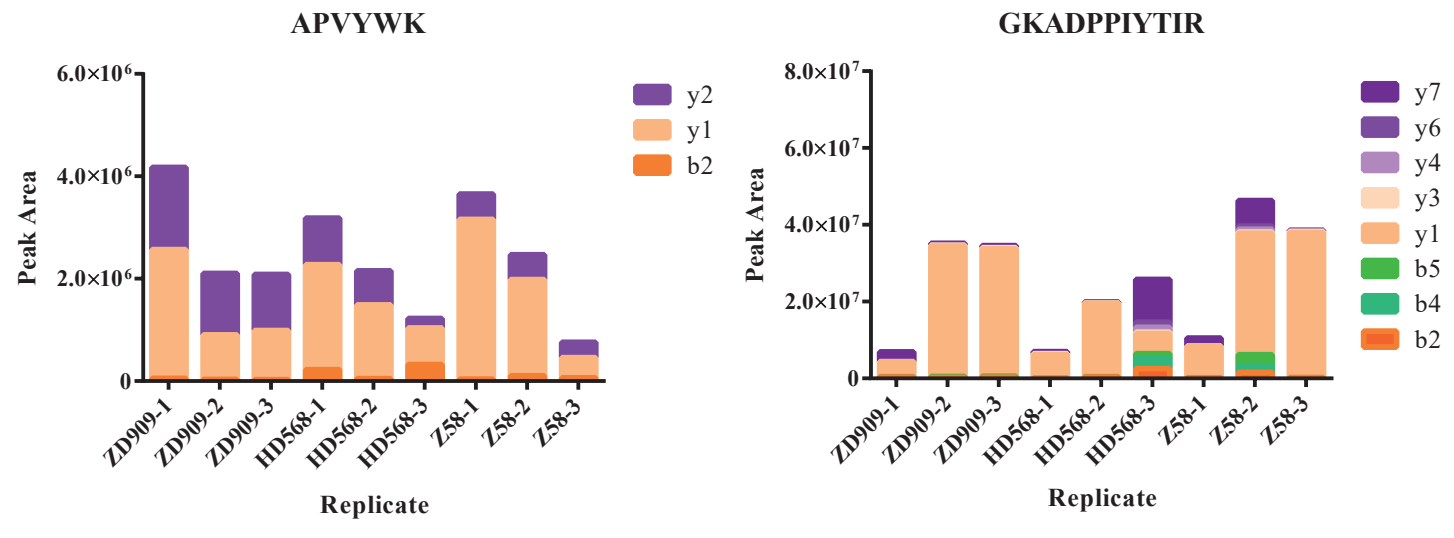

CAB1

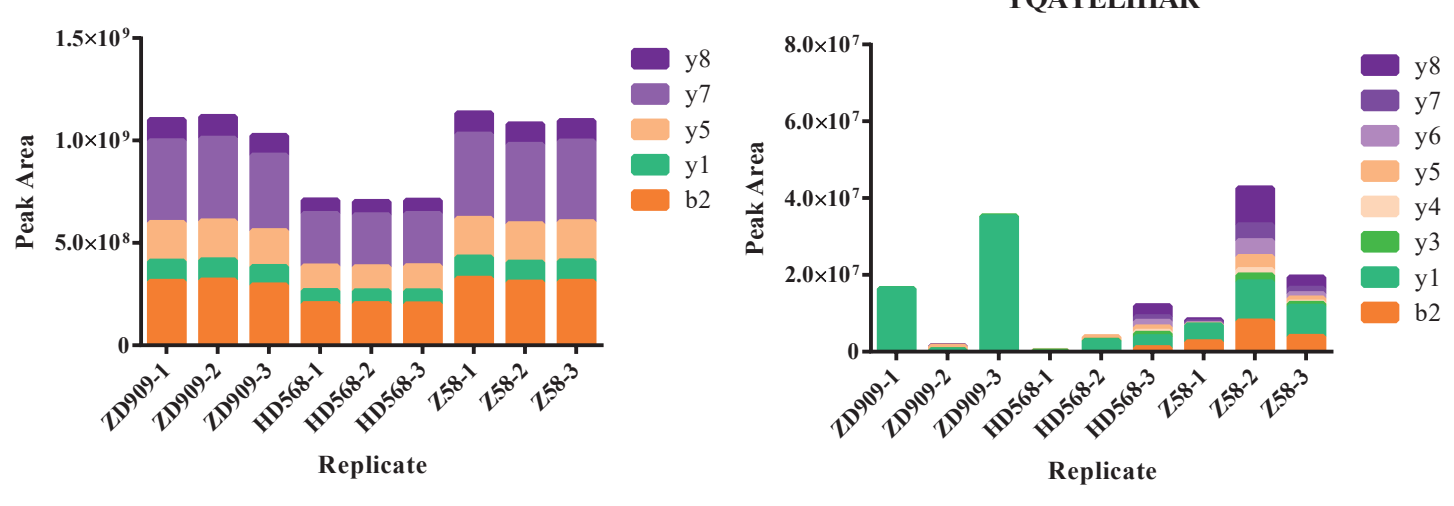

psaB

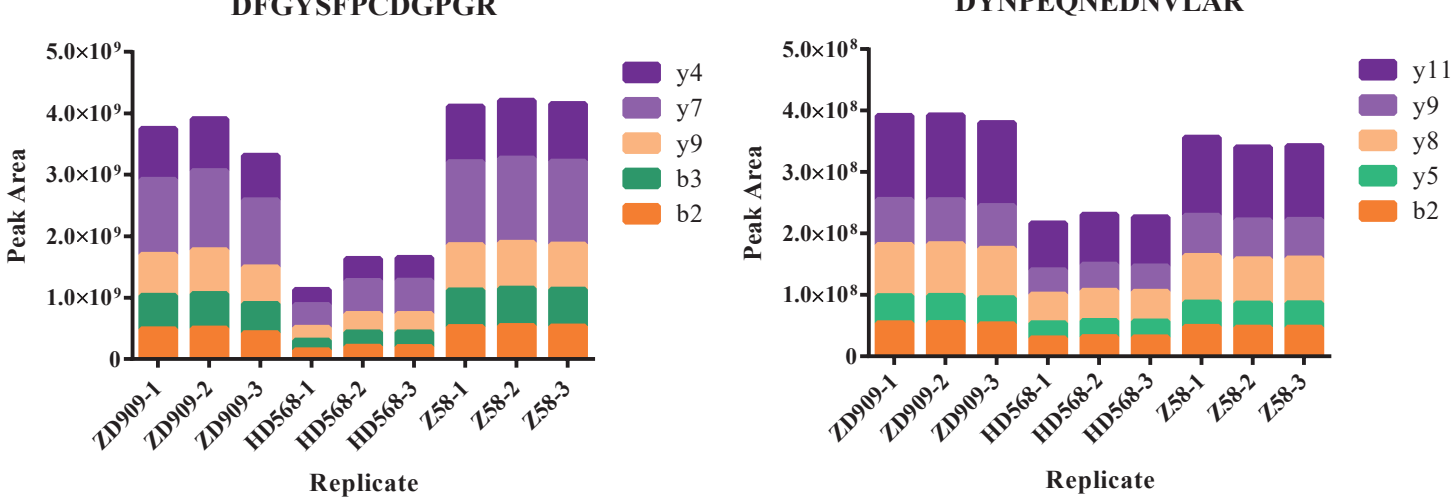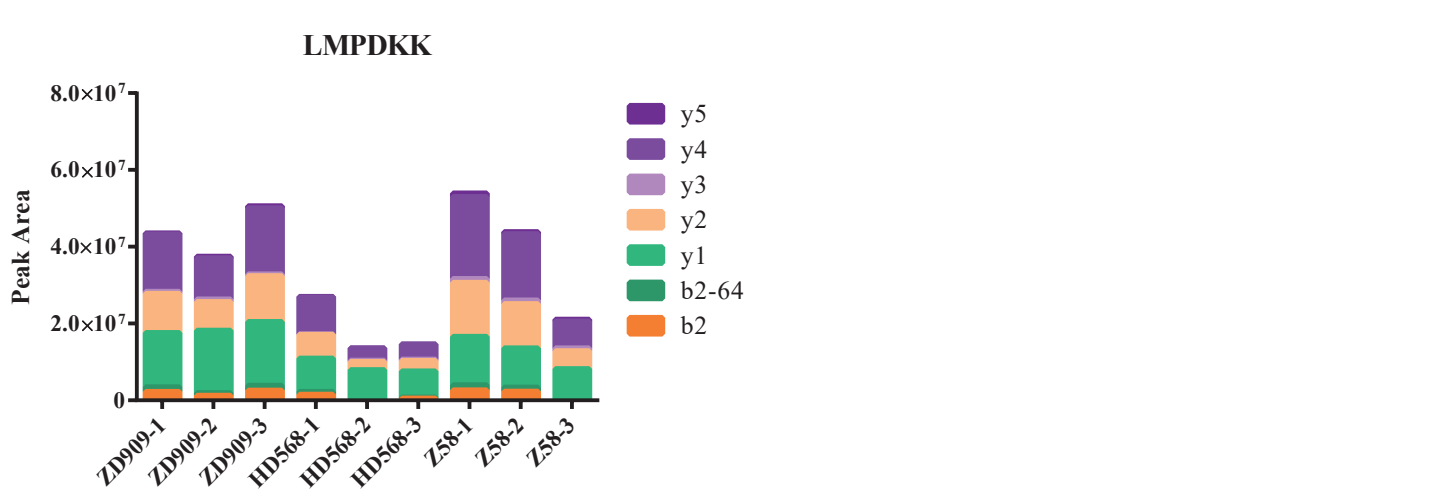

CAB2

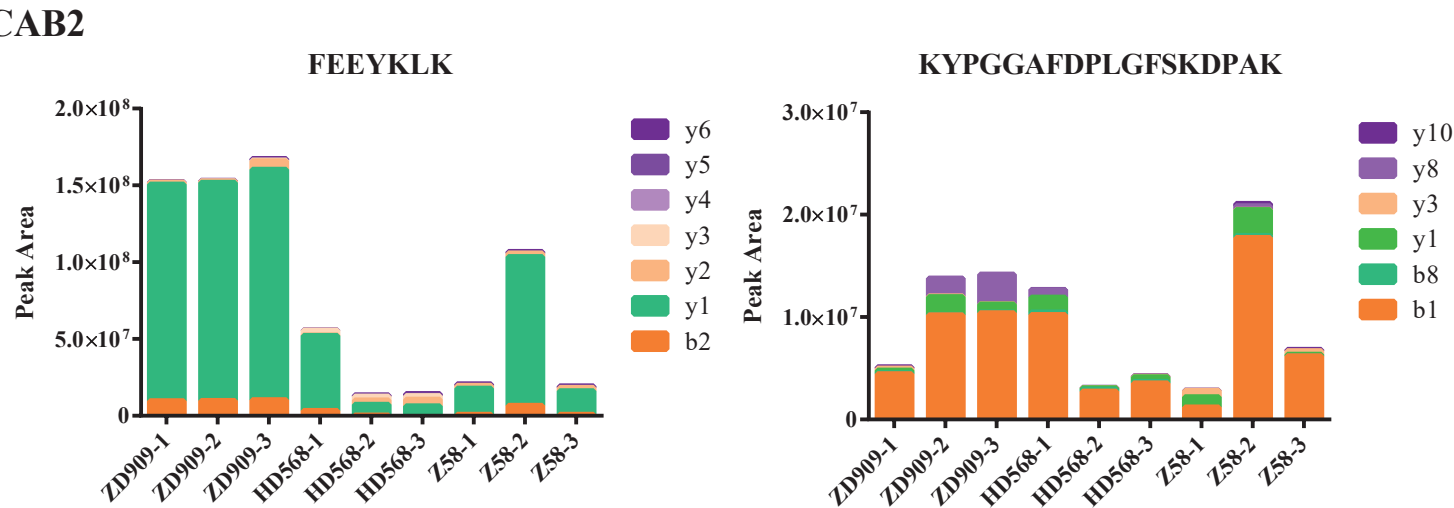

PNSL1

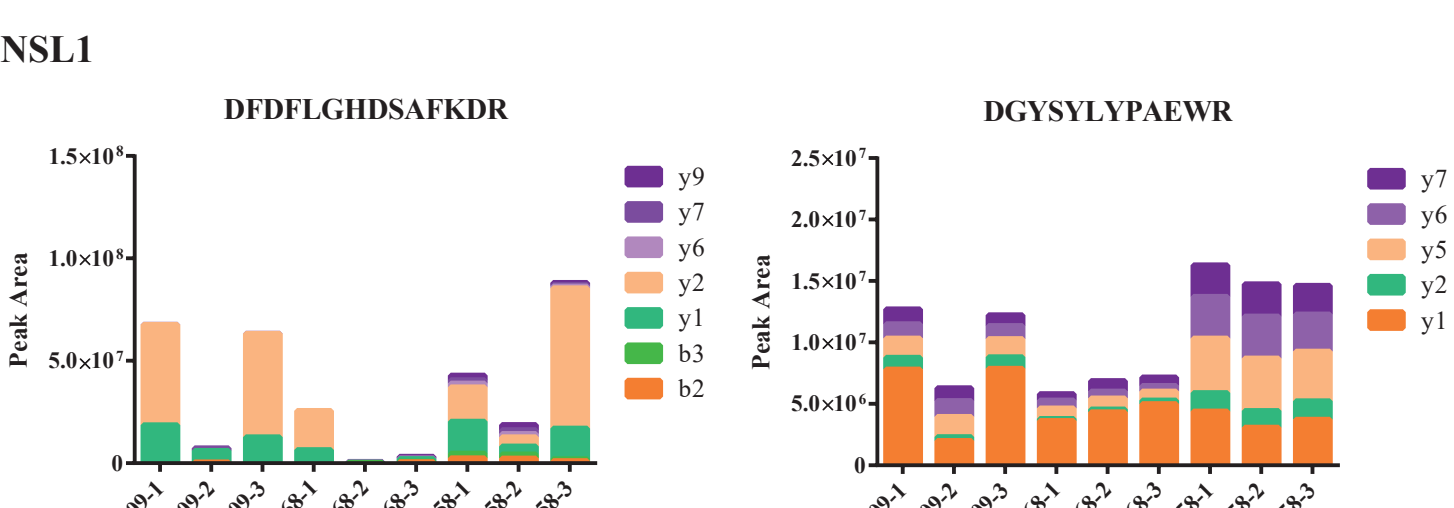

MSBP1

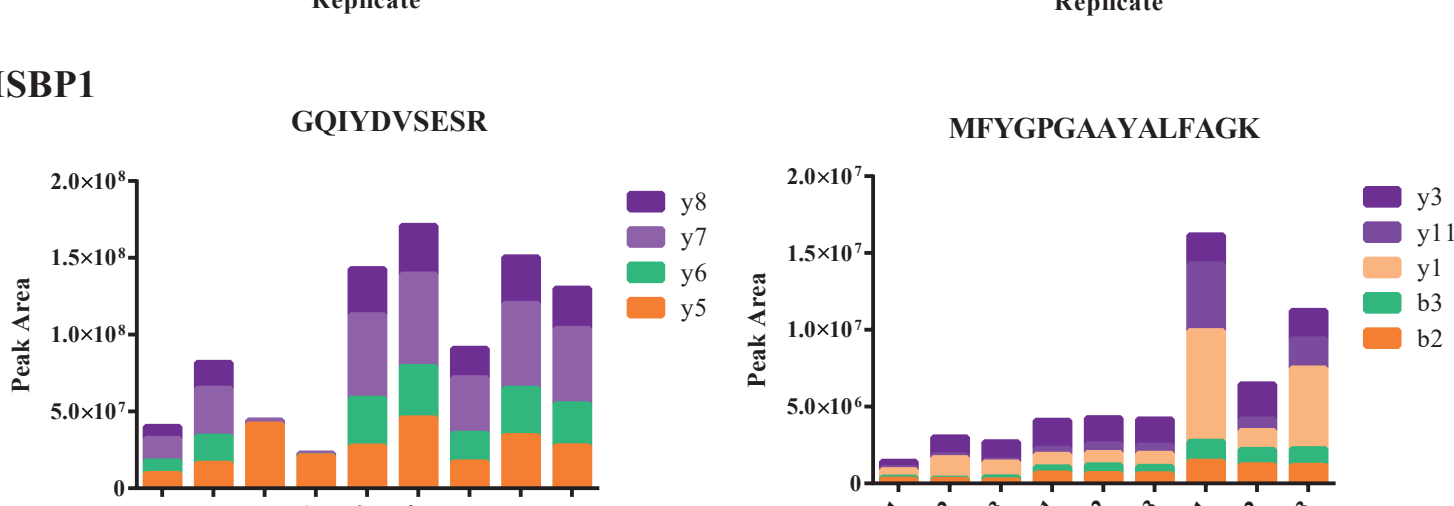

Supplement: Supplementary file 9 — Additional file 9 Fig. S9. Skyline analysis results of 6 PRM verified proteins unique peptides in ZD909. [file 12870_2020_2806_MOESM9_ESM.pdf]
