# Supplementary material for: Comparative proteomic analysis reveals that the Heterosis of two maize hybrids is related to enhancement of stress response and photosynthesis respectively
Source: BMC Plant Biol. 2021 Jan 9;21:34. doi: 10.1186/s12870-020-02806-5 (PMC7796551; doi:10.1186/s12870-020-02806-5)
Supplement: Supplementary file 8 — Additional file 8 Fig. S8. Skyline analysis results of 6 PRM verified proteins unique peptides in ZD808. [file 12870_2020_2806_MOESM8_ESM.pdf]

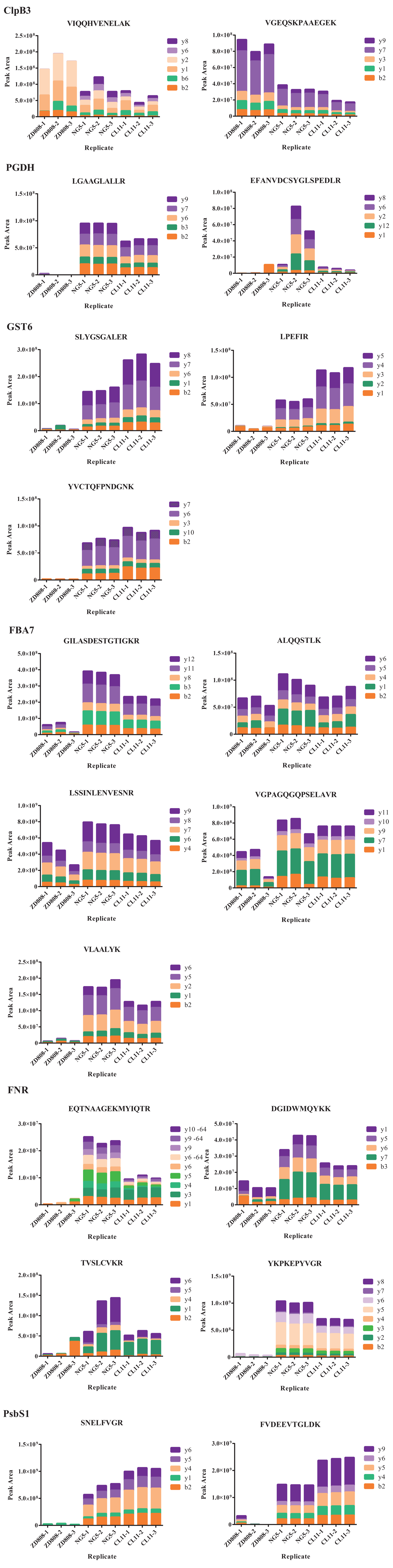
ClpB3
VIQQHVENELAK
VGEQSKPAAEGEK
PGDH
LGAAGLALLR
EFANVDCSYGLSPEDLR
GST6
SLYGSGALER
LPEFIR
YVCTQFPNDGNK
FBA7
GILASDESTGTIGKR
ALQQSTLK
LSSINLENVESNR
VGPAGQGQPSELAVR
VLAALYK
FNR
EQTNAAGEKMYIQTR
DGIDWMQYKK
TVSLCVKR
YKPKEPYVGR
PsbS1
SNELFVGR
FVDEEVTGLDK
Peak Area
Replicate
